# GA-MADRID: design and validation of a machine learning tool for the diagnosis of Alzheimer’s disease and frontotemporal dementia using genetic algorithms

**DOI:** 10.1007/s11517-022-02630-z

**Published:** 2022-07-19

**Authors:** Fernando García-Gutierrez, Josefa Díaz-Álvarez, Jordi A. Matias-Guiu, Vanesa Pytel, Jorge Matías-Guiu, María Nieves Cabrera-Martín, José L. Ayala

**Affiliations:** 1 Departments of Neurology, Hospital Clinico San Carlos, San Carlos Research Health Institute (IdISSC), Universidad Complutense, Madrid, Spain; 2 Department of Computer Architecture and Communications, Centro Universitario de Mérida, Universidad de Extremadura, Mérida, Spain; 3 Department of Computer Architecture and Automation, Universidad Complutense, Madrid, Spain

**Keywords:** Alzheimer’s disease, Frontotemporal dementia, Neurodegenerative diseases, Machine learning, Artificial Intelligence

## Abstract

Artificial Intelligence aids early diagnosis and development of new treatments, which is key to slow down the progress of the diseases, which to date have no cure. The patients’ evaluation is carried out through diagnostic techniques such as clinical assessments neuroimaging techniques, which provide high-dimensionality data. In this work, a computational tool is presented that deals with the data provided by the clinical diagnostic techniques. This is a Python-based framework implemented with a modular design and fully extendable. It integrates (i) data processing and management of missing values and outliers; (ii) implementation of an evolutionary feature engineering approach, developed as a Python package, called **PyWinEA** using Mono-objective and Multi-objetive Genetic Algorithms (NSGAII); (iii) a module for designing predictive models based on a wide range of machine learning algorithms; (iv) a multiclass decision stage based on evolutionary grammars and Bayesian networks. Developed under the eXplainable Artificial Intelligence and open science perspective, this framework provides promising advances and opens the door to the understanding of neurodegenerative diseases from a data-centric point of view. In this work, we have successfully evaluated the potential of the framework for early and automated diagnosis with neuroimages and neurocognitive assessments from patients with Alzheimer’s disease (AD) and frontotemporal dementia (FTD).

**Graphical abstract:**

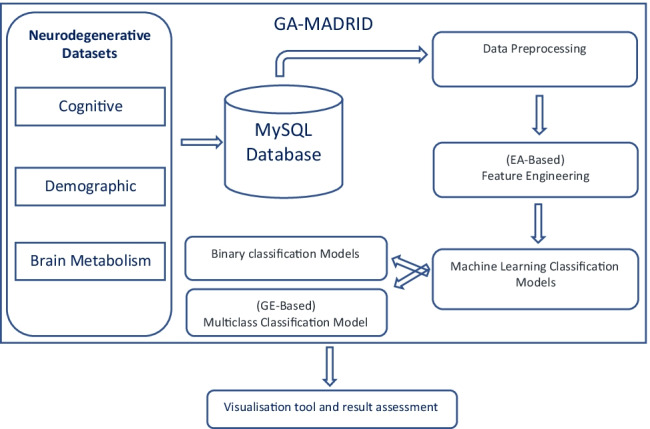

**Electronic supplementary material:**

The online version of this article (10.1007/s11517-022-02630-z) contains supplementary material, which is available to authorized users.

## Introduction

Artificial Intelligence (AI) provides innovative solutions to solve complex real-world problems. Machine learning (ML) is one of its most representative branches with the fastest growing. The health sector frequently generates a large volume of highly dimensional data as those produced by neuroimaging techniques, such as magnetic resonance imaging (MRI) and positron emission tomography (PET) [10]; ML algorithms help on providing diagnosis, decisions, or even predictions related to the health status of patients.

Adjusting the hyperparameters of ML algorithms to get the best performance is not a trivial task; it requires expertise [36]. Therefore, ML models need to be endowed with explainability and transparency on the basis of the eXplainable Artificial Intelligence (XAI) paradigm [28], which will generate confidence and reliability in the results. This fact is connected to AI democratization [35] and the open science perspective, where sharing and collaborating are two essential objectives.

The interest of the scientific and medical community in providing solutions based on AI to enhance and assist in the diagnosis, prevention and/or development of new treatments has increased significantly [45]. Despite the assistance, caution is needed to prevent any unintended though negative consequences that may occur, for instance if some data are not contextualised [9].

Mentioning some scientific literature in this domain, [45] analysed the potential of AI and ML for the medicine field, and identified changes and challenges to reach accurate and comprehensive diagnosis. [1] presented a review of different solutions, approaches and perspectives of AI and ML, especially for the healthcare sector. Authors included a critical vision, where they pointed out some issues to be improved in order to guarantee the privacy and data security, and to enhance accuracy. Recently, [52] provided a review on current computational approaches applied in the spectrum of neurodegenerative diseases.

Focusing on neurodegenerative diseases and *Python*-developed studies, [29] performed a ML-based analysis to perform data-driven diagnosis of dementia and used post-mortem confirmed cases as a gold-standard; [43] implemented a pipeline based on a DeepSymNet architecture to detect the AD progression pattern. Recently, [53] applied the feature engineering to build voice biomarkers and improve the early detection of Parkinson disease. [8] analysed a group of individuals diagnosed with both behavioural and language variants FTD, using a deep learning algorithm. [17] assessed ^18^F-2-fluoro-2-deoxy-D-glucose positron emission tomography (18F-FDG PET) brain images from Alzheimer’s Disease Neuroimaging Initiative (ADNI) dataset and a retrospective independent test set through a convolutional neural network of InceptionV3. [14] tackled the classification of Alzheimer’s disease into four classes using 3D Diffusion Tensor Imaging (3D-DTI) processing.

Neurodegenerative diseases include a wide spectrum of disorders with different clinical manifestations and pathological patterns, where an early accurate diagnosis is challenging. AD is one of the most prevalent [20] and causes a progressive and irreversible brain damage that prevents patients from performing daily activities. FTD is the third cause of dementia, particularly the behavioral variant (bvFTD), and its onset occurs at middle-age [20]. In this work, we specifically focus on AD and FTD, although other neurodegenerative diseases could be similarly addressed by our proposal.

The assessment of patients who suffer from neurodegenerative disorders entails the application of neuroimaging techniques, neuropsychological tests and the clinical histories [23]. Neuropsychological tests assess the cognitive function affected by AD and FTD. Among the neuroimaging techniques, 18F-FDG PET is a minimally Invasive technique. 18F-FDG PET gives a map of brain coordinates associated to metabolism rates, which measure the alterations of glucose consumption in the brain. The presence of alterations in brain metabolism has proven to be a useful biomarker for early diagnosis of AD and FTD [7, 26, 39]. These techniques provide a large volume of data [10], which require experts to be trained in their analysis and interpretation, but the risk of inaccurate diagnoses is real, especially considering the need of early detection of these disorders [18]. In this context, ML techniques are a reliable alternative for design decision-making models that support specialists in the early diagnosis of the disease, monitoring and designing personalized treatments [45], where accuracy is extremely important.

Last decade, many researchers have demonstrated their potential for supporting decisions-making in the clinical arena [11, 25, 34]. However, to the best of our knowledge, we cannot find any other framework in the literature that targets the fully automated diagnosis of AD and FTD from multiple and heterogeneous data sources. The proposed computational tool embodies all the required steps to deal with the data modelling process.

Thus, it integrates the following functionalities:

1. An automate methodology for dataset preprocessing, including imputation techniques to deal with missing, outliers and categorization of nominal variables.
2. A feature engineering module implemented by means of evolutionary algorithms to extract the most relevant features for the diagnosis.
3. A meta-model based on evolutionary grammars and Bayesian networks (BN) for multi-class classification.
4. A basic visualization tool.
5. Different tools for assessing the results.

This work is structured as follows. Firstly, the framework designed is presented. Subsequently, results of the tests using the proposed AI-based tool are summarized and the following section discusses the results of the test case. Next, the conclusion are presented. Finally, the general methodology is described.

## Methods

**Fig. 1:**
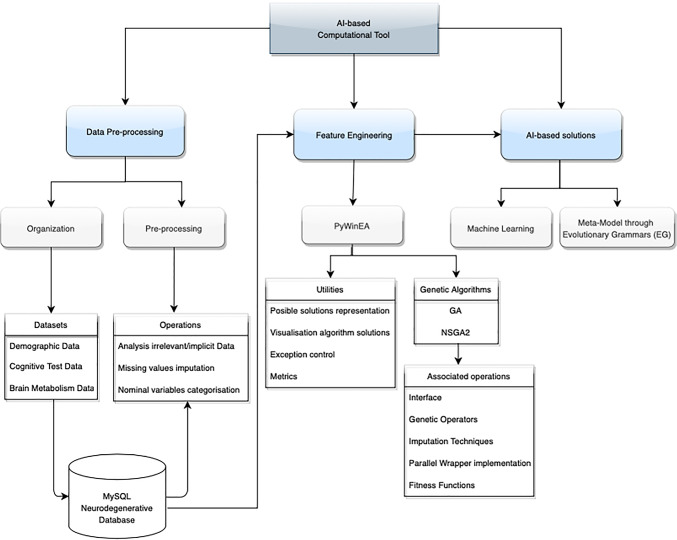
General scheme of the AI framework proposed, including data pre-processing, feature engineering and IA-based modelling

This is a Python-based framework that makes the data modelling easier to be computed and it is fully extendable thanks to its modular design from the data-driven point of view. According to the general scheme presented in Fig. 1, this Python-based framework provides resources to address data pre-processing, feature selection, a wide set of machine learning models, different AI-based modelling strategies with mono-objective and multi-objective evolutionary algorithms. It also implements a multiclass classification model using EG or Bayesian classifiers. In addition, it provides graphical evaluation tools based on different metrics to asses the results obtained.

Focusing on supplying a fast, robust and reliable AI-based tool, the proposed framework is able to deal with different datasets, including cognitive evaluation, neuroimaging techniques, and the patients’ history to help in the diagnosis of AD and FTD, two neurodegenerative diseases that may present similar symptoms and cognitive and behavioral deficits. Although episodic memory dysfunction is one of the cognitive hallmarks of AD, FTD usually presents also these symptoms. Similarly, behavioral deficits are increasingly recognized symptoms in AD [24, 41]. The management and organisation of data are carried out through a relational database, particularly MySQL. Data are structured in indexed tables that ensure the accessibility, availability and simplifies the data preprocessing. Additionally, relational databases and processes are implemented to easily incorporate new data and guarantee the data integrity.

As aforementioned, this framework manages three types of data: (1) Demographic Data provide variables that describe the sample; (2) Cognitive Test Data contain variables associated with cognitive tests, where each cognitive test provides a rating scale and scores to identify specific kind of cognitive problems and abilities. These tests gather information about the following cognitive function: memory, visuospatial, executive, attention and language. (3) Brain Metabolism Data include the brain hypometabolism data from the FDG-PET analysis.

Regarding the brain regions, this framework considers two different atlases, the Brodmann’s atlas (47 regions)[6] and the Automated Anatomical Labelling (AAL) atlas (90 regions)[54]. Data related to the brain metabolism are divided into qualitative and quantitative. Quantitative data are defined by the number of hypometabolic voxels in a given region. A voxel is a 3D unit of an image, which can be associated with a single value, such as metabolism. Hypometabolic voxels are computed through the voxel-based mapping analysis against a healthy control group. The qualitative data indicate whether a certain area is hypometabolic or not. Although the number of voxels needed to consider a regions as hypometabolic may vary, in this study we selected a threshold of 1 voxel in each region. Therefore, a region was defined as hypometabolic when it has one or more hypometabolic voxels. Although we agree that this is a very limiting threshold, the purpose of this work is to present a parameterizable computing framework, in which this threshold, as many other parameters, can be selected by the expert user in order to meet its clinical goals. The clinical value of the results obtained by the use of our proposed framework is out of the scope of this publication, but is has been already proven in [27].

In order to reduce the effort to reproduce experiments, adapt the implementation to the XAI perspective and gain trust and reliability, both data and the developed script to process data are available on https://github.com/greendiscbio/neuro_MiningAndModeling/tree/Diagnostic_aid_model on request from computational and clinical researchers 1, where all necessary explanations are provided.

The aim of this work is the development of the computational framework, which is widely customisable and scalable. In this publication, we do not target the accuracy of the clinical assessment provided by the tool and presented in publications like [27], but we discuss around a case of study to show the functionalities of the AI-based tool.

This computational framework has been evaluated using a dataset, which includes cognitive and PET data from 329 patients (171 AD, 72 bvFTD and 87 Healthy controls. As this work is focused on the presentation of the computational framework, we use our own dataset because the data labelling is controlled. Although, this framework has been designed to be able to work with publicly available datasets. This comprehensive dataset is structured using different combinations in order to present a comprehensive and consistent study. Patients included in this study had a neuroimaging compatible with FDG-PET meeting the current diagnostic criteria [2, 38, 46]. The diagnosis was confirmed after over two years of follow-up. Spouses and volunteers were recruited as Healthy Controls meeting the following criteria: (1) absence of cognitive impairment, according to a MMSE score ≥ 27 and Clinical Dementia Rating of 0 (Morris, 1993); (2) absence of functional impairment measured by Functional Activities Questionnaire scores of 0 [40]. The exclusion criteria were as follows: (1) prior or current history of other neurological diseases (e.g. stroke, brain tumour, seizures); (2) history of psychiatric disease, alcohol or psychotropic drugs abuse; (3) visual, hearing, or any physical problem with a negative impact on test performance.

Regarding data, the Institutional Research Ethics Committee from Hospital Clinico San Carlos approved the research protocol with the 1964 Helsinki declaration and its later amendments. Informed consent was obtained from all individual participants included in the study or their caregivers.

Once the dataset is defined the data preprocessing and feature selection tasks are carried out. Subsequently, AI-based modelling strategies can be launched, and finally, analysing the results obtained through the available metrics in this framework.

## Results

This section presents the framework design. The code is made available through the *GitHub* and *Pypi* platforms. Figure 1 represents the general scheme of the proposed framework, which is divided into three different parts: (i) Data pre-processing, (ii) Feature engineering, and (iii) AI-based modelling.

### Data pre-processing

Considering the specifications in Section 2, the database has been structured according to the following layout, each brain atlas has two associated tables, one with hypometabolism quantitative data and the other with qualitative data. On the other hand, cognitive evaluations are subdivided into screening and specific tests. Within the specific test there are either raw scores (specific_raw) or scores corrected according to gender, age and years of education (specific_corrected).

Data pre-processing includes all the tasks described below.

#### Data cleaning

This task analyses data and eliminates variables that are neither irrelevant o implicit in the data. Thus, PET date, date of birth, age of disease onset, date of visit, read/write and Mini Mental State Examination (MMSE) were excluded. It also examines the brain data for inconsistencies or incoherencies, e.g. 9 instances with normal brain metabolism which are classified as AD or FTD patients. This information is presented to the user in order to request an action on those instances and/or variables..

#### Processing of missing values

This task is responsible for identifying empty values from the available dataset. It also handles the missing data imputation task. The applied imputation techniques depend on each given dataset and prediction model to be used, so its applicability to another dataset should be analysed. In this framework, missing values imputation was carried out using the non-parametric MissForest imputation technique [50], which is able to identify non-linear and complex relationships between variables. MissForest is an extension of the MICE methods that apply a multivariate and iterative imputation [4], and gives more realistic results than other parametric techniques [50].

#### Categorization of nominal variables

This task is responsible for applying encoding techniques to nominal variables. One Hot Coding is the most frequently used coding scheme, which transforms a single variable with “n” different values into “n” binary variables. Each binary variable represents a single value and the presence is indicated with a 1 and the absence with a 0.

Since the first step of the analysis consisted of a selection of characteristics and each variable in the one hot vector represents a new characteristic, it was not necessary to remove a variable to avoid multi-collinearity problems.

### Feature selection

In high dimensionality problems, identifying the most relevant attributes is a crucial step when modelling data through ML and the problem is an NP problem [13]. Reducing the dimensionality enhances interpretability, a key aspect under the XAI perspective, makes clinical diagnosis easier, improves the performance of classification models, reduces the computational cost and prevents the models from overfitting [48]. This task aims to remove irrelevant and overlapping features from the whole set of features, while retaining the most relevant ones. Hybrid approaches using wrapping techniques, and heuristic and metaheuristic search strategies [32, 59] are very efficient to explore the feature space. Feature selection via evolutionary algorithms [58], as one of the most popular metaheuristic, is selected for the implemented computational tool.

This AI-based tool integrates the feature selection through the **PyWinEA** module, a *Python* package developed on the top of the scikit-learn library that implements the most widely used genetic algorithms. This module is capable of working with data provided by current evaluation and diagnostic techniques. **PyWinEA** package has been endowed with a basic GA and MOEA (NSGAII) to explore the feature space. These techniques and their use along this work are introduced below.

#### Evolutionary algorithms

Evolutionary algorithms (EA) are population-based techniques inspired by the process of natural selection. They evolve a population of individuals, that represent potential solutions. Individuals will experiment variations to simulate the genetic changes, which guide the evolutionary process.

EAs show a high exploratory capacity, including discontinuous search spaces with a lower tendency for local maxima. This work considered a maximization problem given the interest in improving the models performance. The *PyWinEA* package defines the genotype of the individual as an array of integer values of variable length. Each integer represents an attribute, and the mapping process consists of substituting the integer with the values associated with the attribute. The fitness function is given by the classification model and its classification performance. *PyWinEA* implements two stochastic selection operators: fitness proportional selection and tournament selection, and two survivor selection strategies: elitism and annihilation. Finally, the mutation and recombination operators, random resetting and one-point crossover, are implemented as variation operators.

#### Multiobjective evolutionary algorithms

Most real problems require more than one metric to evaluate the quality of a potential solution. Frequently, there are several objectives to maximize and usually, they are conflicting objectives. The optimal solutions in multi-objective optimisation deal with the domination concept, which determines the non-dominated front of solutions also called Pareto’s front [19, 195–198]

Consequently, when there are two objective functions that are contradictory (e.g. the classification performance and the number of characteristics in the subset), a unique solution may not dominate the rest. In this situation, we are interested in finding the set of non-dominated solutions that are closest to the optimal Pareto’s front.

One of the most used MOEAs is the NSGAII [15], which has been implemented in *PyWinEA*. Solutions in the optimal Pareto’s front were evaluated by the hypervolume indicator (*I*_*H*_), which has been applied using the inclusion-exclusion algorithm [57]. *I*_*H*_ is a unitary measure defined in [5] as “the d-dimensional volume of the hole-free orthogonal polytope”.

**Fig. 2:**
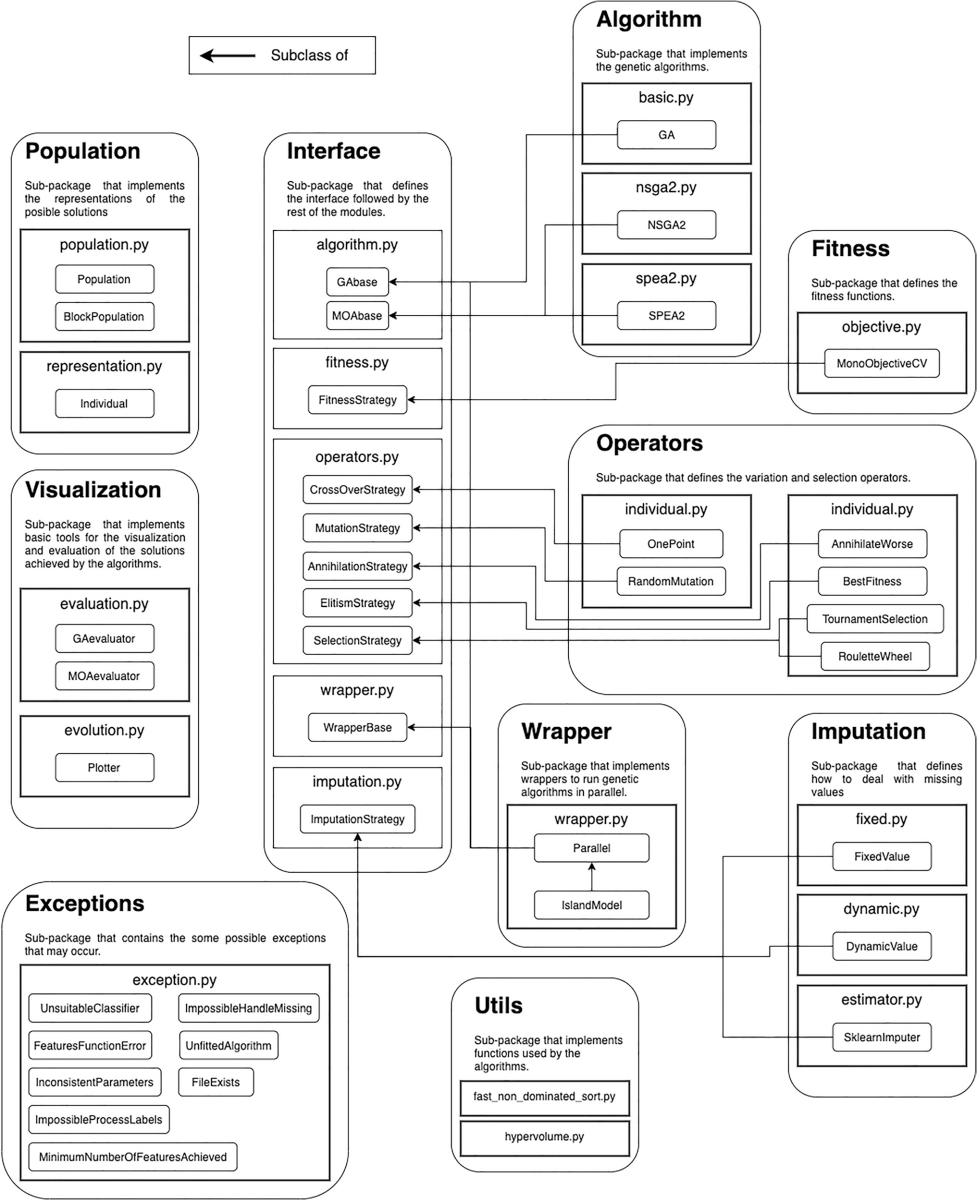
Structure of the PyWinEA package used for feature selection This package is available through PyPi and GitHub

A set of supervised classification algorithms has been used to evaluate the quality of the solutions in the feature selection process implemented in *PyWinEA* Fig. 2, and to develop (ML)-based solutions.

### ML-based solutions

This section presents the methodology used in developing several learning models to assist clinicians in the diagnosis of AD and FTD.

#### Machine learning models

This computational tool integrates several classification models to provide clinicians with a widely comparative framework. In this light, different classifiers and their performance can be analyzed using the features selected by the EA algorithm approach. Although any parameter of the classification algorithms can be adjusted, for each classifier we only mention the most significant ones when addressing this particular problem.

- **Bernoulli naive Bayes**. This model allows to adjust the prior probabilities of each class and the smoothing of the variance.
- **Support Vector Machines**. The RBF (Radial Basis Function) was used as a kernel function and the *γ* and *C* parameters were adjusted.
- **K-Nearest Neighbors**. Different number of neighbours and distance metrics were explored.
- **Decision Trees**. Alternative ways of partitioning the nodes (using the best split given by the Gini criterion or by randomly partitioning the nodes), the maximum depth, the minimum number of samples in each split and the minimum number of samples to declare a node as a leaf were the adjusted hyperparameters.

In addition, three ensembles based on decision trees were used. For each one, the number of base estimators and their hyperparameters were tuned:

- **Random Forest**.
- **AdaBoost**. Different learning rates were considered.
- **Gradient Boosting**. The learning rate, the fraction of samples used to train each of the estimators as well as the loss function were adjusted.

Four functionalities were also developed: (1) Graphical evaluation using training and validation; (2) General functionalities such as loading datasets and exception control; (3) Graphical representations among several classification models; (4) Performance evaluation using accuracy, F1-score, precision, recall, sensitivity and specificity metrics, as well as receiver operating characteristic (ROC) curve and classification errors.

Using these functionalities, every classification model provides graphical resources to evaluate the performance of the results, thus the confusion matrix, accuracy, F1-score, precision, recall, learning rate, sensitivity, specificity, the area under Receive Operating Characteristics (ROC) curve and the classification errors are graphically represented.

The proposed classifiers cover most of the problems that can be defined with the data processed in the Section 3.1 section. Moreover, a new multiclass classification strategy for cognitive tests is described below.

#### Meta-model strategy

This work explores a new high quality strategy to improve the classification performance especially designed for cognitive tests when tackling *One vs Rest* problems. It integrates the information provided by each binary classifier into a multiclass single model.

**Fig. 3:**
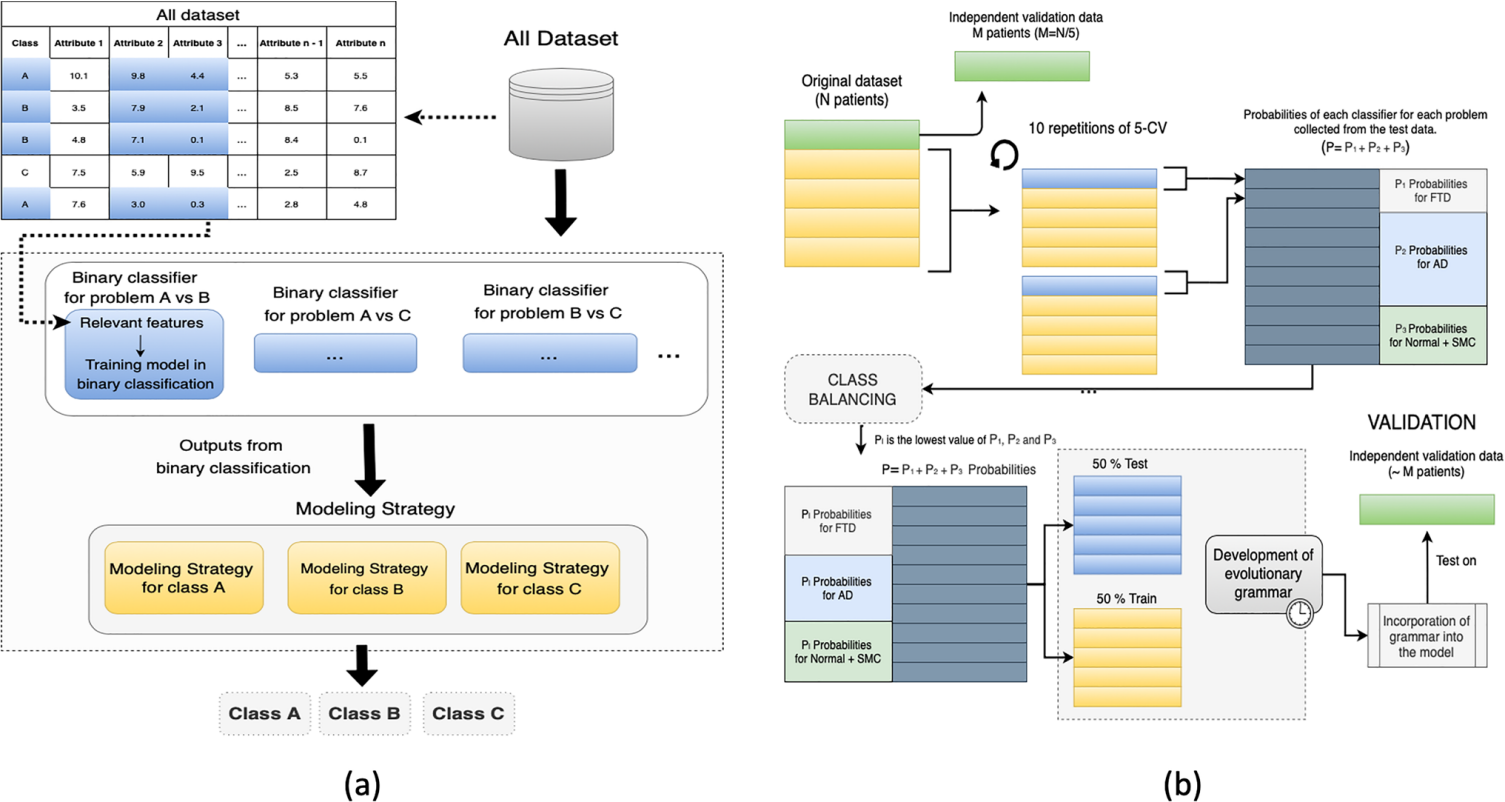
Meta-model scheme considering a problem with three classes *A*, *B* and *C*. The modeling strategy takes the output of the binary classifiers of the previous layer and the class assigned to an example will be the one with the highest value

The proposed meta-model is a two-layers design, as presented in Fig. 3, according to a stacking strategy [56]. The first layer is responsible for the binary classification, operating in a different feature space and using characteristics selected during the feature engineering process. This layer uses SVMs as binary classifiers and forwards their results to the second layer that generates a multiclass output. The second layer applies a modeling strategy based on evolutionary grammars or Bayesian networks. In this model, each of the binary classifiers of the first layer operates in a different feature space. Features selected during the feature selection phase were used. Additionally, every binary classifier was trained using different examples, which were driven by the binary problem addressed.

Every target class is associated with one or more binary classifiers. The classification process generates a probability or binary value, which indicates the class that a sample belongs to. The modeling strategy associates the results of the binary classifiers to a single real value. The highest value will identify the final class.

Figure 3 considers a problem with three classes *A*, *B* and *C* and three binary classifiers *C*_*AvsB*_, *C*_*AvsC*_ and *C*_*BvsC*_, which use different characteristics to perform the classification. Given a training dataset *T*, the first step consists on the generation of three datasets *T*_1_, *T*_2_ and *T*_3_. The dataset *T*_1_ associated with *C*_*AvsB*_ is composed of the characteristics selected for the *A* vs *B* problem and the examples labelled with classes *A* and *B* excluding the examples belonging to *C*. The same is applied to datasets *T*_2_ and *T*_3_.

During the prediction phase, we will have a modelling strategy associated to each class. The modelling strategy associated to class *A* will receive the outputs of classifiers *C*_*AvsC*_ and *C*_*AvsB*_, the same for the rest of the classes and their associated classifiers. The class selected will be decided upon the modelling strategy that provides the highest value.

##### Evolutionary grammars as a modelling strategy

Evolutionary grammars (EG) are part of EAs and an approach to genetic programming. Solutions are generated using a grammar representation. EG has obtained promising results in many domains such as the prediction of migraine crisis [42] or glucose levels [12, 30, 55].

**Fig. 4:**
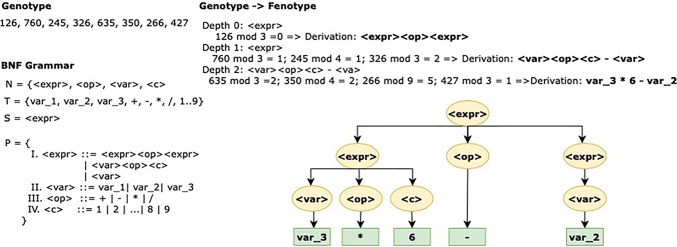
Genotype to phenotype mapping process following the syntax described in the grammar. The next node to be chosen during the mapping process is determined by the genotype codon module and ends when a terminal node is reached

Representing the genotype with an array of integer or binary values, the genotype-to-phenotype decoding uses a Backus Naur Form (BNF) grammar [47]. Figure 4 describes an example of the mapping process. A grammar is represented by the tuple {*N*,*T*,*P*,*S*} where *N* and *T* are the non-terminal and terminal symbols, respectively; *P* are the production rules applied to generate *T* from *N*, and *S* is the initial expression. The result is a tree structure where S represents the root, N the intermediate nodes, P the potential paths and T the leaves.

**Fig. 5:**
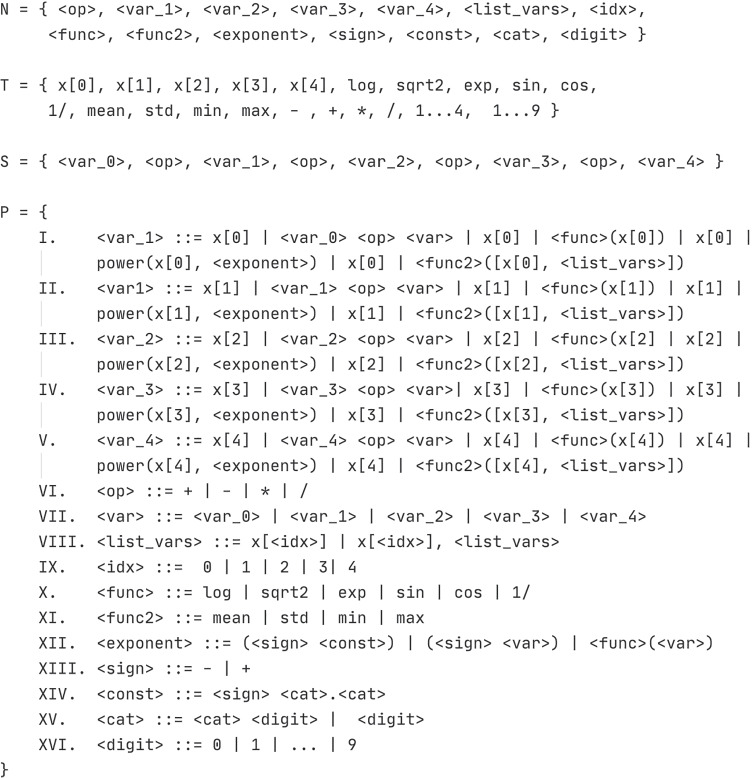
Grammar to handle the genotype to phenotype mapping process

Figure 5 shows the grammar used to define the geno-type-to-phenotype mapping process, where the gender and age variables are not included to avoid bias. The variable *x* refers to the set of predictions made by the binary classifiers of the previous layer, therefore the index indicates the position of the output of the algorithm associated with a given binary problem.

**Fig. 6:**
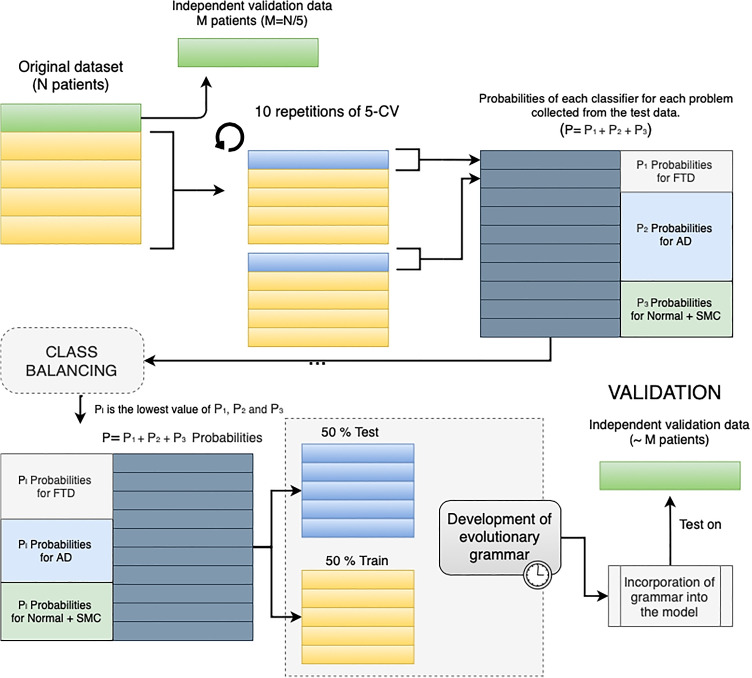
Methodology designed for the development and validation of EG as modeling strategy. A class stratification following a CV scheme was implemented. If classes were imbalanced, classes would be balanced to the minority class

Figure 6 shows the methodology followed by the proposed meta-model using EG as modelling strategy. The steps are described below:

1. The dataset was divided into 5 disjunct datasets with class stratification following a cross-validation (CV) scheme.
2. One of the datasets is reserved independently for the validation process. With the remaining four, the binary classification phase is launched for 10 iterations with a 5-CV scheme. The predictions of the binary classification models generate a new dataset.
3. If classes were unbalanced, at this point they would be balanced to the minority class. This is carried out by randomly removing predictions from the majority classes until all classes are balanced.
4. The grammar development uses 50% of the dataset samples for training and 50% for testing. This process can be defined as a new supervised classification problem
5. This grammar is integrated into the model as a modelling strategy. The validation of the meta-model is performed with the independent dataset from the step 1.
6. The steps from 2 to 5 are repeated for each of the 5 separate folds in step 1.

The described procedure allows to make an approximation of the generalization capacity of the meta-model that incorporates EG in the second layer. Based on the approach of [44] and given its influence on AD [3], the methodology described was repeated after introducing the gender and age variables into the prediction dataset and including them in the grammars. Thus, the production rule ***VII*** was modified to include the gender (x[5]) and age (x[6]) variables: 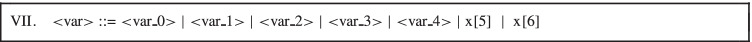


The grammar was implemented using the Python package PonyGE2 [22]. Table 1 shows the default selected parameters, although other parameter values can be applied.

**Table 1:** Parameters used for the development of evolutionary grammars using PonyGE2

Parameter	Parameter setting
Algorithm	NSGAII [15]
Population size	300
Elite size	30
Generations	1500
Crossover	subtree^a^
Crossover probability	0.9
Mutation	subtree^a^
Mutation events	1
Selection proportion	0.5
Fitness 1	F1
Fitness 2	Minimizing the number of nodes
Maximum derivation tree initialization depth	10
Maximum derivation tree depth	15
Initialization strategy	PI grow [21]

^a^ The crossover strategy is analogous to the one-point operator but by mixing tree structures. The mutation operator is applied only to the population resulting from the crossover

##### Bayesian networks as a modelling strategy

Bayesian networks represent a sub-type of probabilistic graphical models. This type of model uses directed acyclic graphs (DAG) to represent the probabilistic relationship between variables. Nodes correspond to variables and an arc between two nodes shows the dependency relationship. In this type of models, every node is associated to a local probability distribution, which is usually specified by a conditional probability table (CPT), and depends on its parents [33, 42–92]. Each node receives an input and gives the probability distribution of the variable associated to the node, as an output 2.

In this computational tool, the meta-model based on Bayesian networks was implemented on the top of Pomegranate library [49]. Each node in the network corresponds to a binary classifier associated with a given problem. Thus, the Bayesian network allows to model the joint probability distribution of the output of the binary classifiers by assigning a probability to each possible combination of outputs. The two steps required to build a Bayesian network include learning the structure and determining the probability distribution associated with each node based on the data. The structure was determined using a score-base approach, applying dynamic programming and the A* algorithm in order to maximize the probability of the data given the model by means of maximum likelihood estimation.

The dataset generated by the grammars during the step 1 was used for the learning network. Predictions were binarized by rounding up to the nearest integer. A Bayesian network was developed to model the joint probability for each of the classes in such a way that, the label assigned to a new example corresponds to the class whose associated Bayesian network, given the evidence (binary classifier outputs), yields the maximum probability.

## Discussion in a case of study

This section presents some outcomes that can be achieved by the proposed framework in a particular case study of neurological diseases: clinical diagnosis of AD and FTD. A description of data in this study is presented in Section 2. Although, this study is not focused on the clinical analysis of AD and FTD by means of the proposed framework, we present a case of study using PET data in order to show the functionalities of the tool. Particularly, data preprocessing phase, feature engineering using NSGAII, classification using different ML algorithms, multiclass meta-model with EG and Bayesian networks, and some of the graphical resources to outline the results. We expect that, with this case of the study, the reader will understand the capabilities of the proposed computing framework and will be able to value the potential of the tool in its clinical practice.

### Data preprocessing

**Fig. 7:**
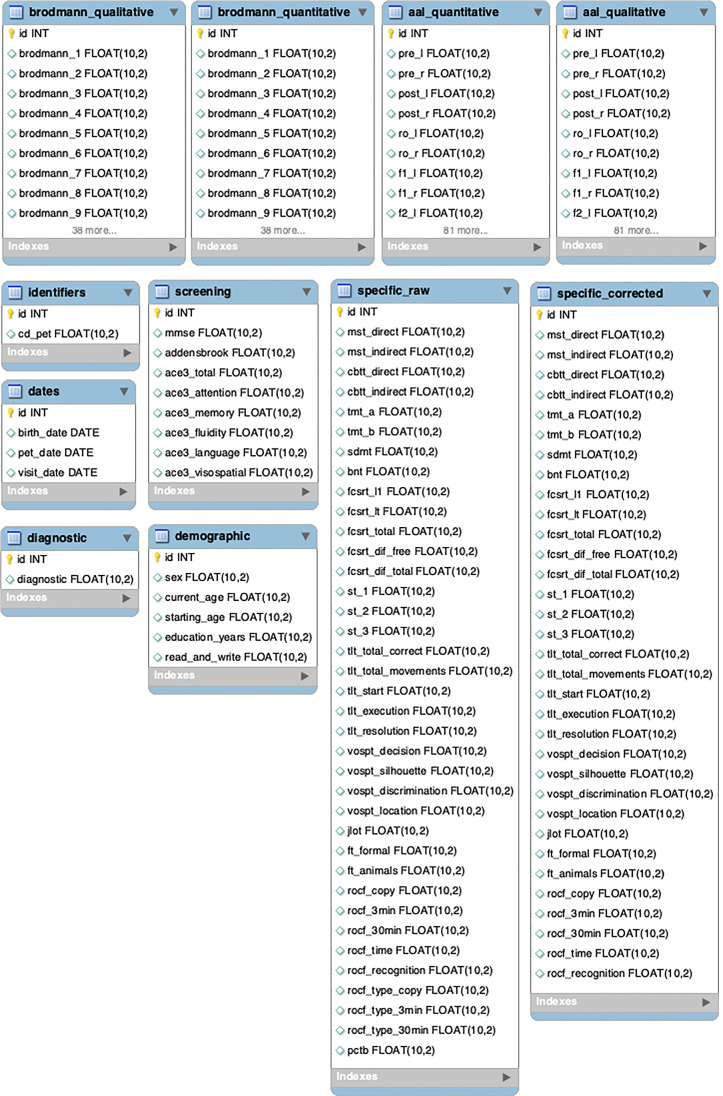
The structure of the database designed. Tables brodmann_qualitative/quantitative and aal_qualitative/quantitative, corresponding to the brain metabolism data have been shortened. Complete data are available on request on GitHub

Regarding the data pre-processing described in Section 3.1, data were structured in a relational MySQL database shown in Fig. 7, which can be extendable as needed.

Once the database was ready, data were analysed within the cleaning process and removed irrelevant data. Then, the analysis of the missing values was carried out, which represented 11.28% in the database. After its identification, the Missforest imputation technique was applied with 100 as the maximum number of interactions and the following parameters settings: Mean for the initial imputation; 1*e* − 03 as early stopping; 50 as number of trees (default parameters for decision trees); Mean squared error as the evaluation criterion of each partition, and random for splitting each node.

The last step was dealing with nominal variables following the methodology presented in Section 3.1. The imputation generates real values, which will be rounded to the nearest integer. Next, the one hot coding scheme is applied and as many variables as different values were added.

### Features engineering

The aforementioned use case was addressed by bi-objective MOEA approach, previously described in Section 3, and a customization of hyperparameters as shown in Table 2, where the two objective fitness function are also described. 10 iterations of 5-CV were run and the performance of the best subset obtained for each classifier was evaluated. The algorithm were run for 10 iterations of 5-CV and the performance of the best subset obtained for each classifier was evaluated.

**Table 2:** Selected parameters for the NSGAII algorithm used to carry out the feature selection

Algorithm parameter	Parameter setting
Mutation strategy	Random Resetting
Selection strategy	Tournament selection^a^
Fitness 1	Accuracy or F1^b^
Fitness 2	Number of features^c^
Number of different initializations	2

^a^ k = 2, winners = 1 without replacement

^b^ 5 repetitions of 5-CV with class stratification

^c^ Defined by equation: $1-\frac{\mathrm{Length}\;(\mathrm{Individual})}{\mathrm{Num}.\;\mathrm{Features}}$

**Fig. 8:**
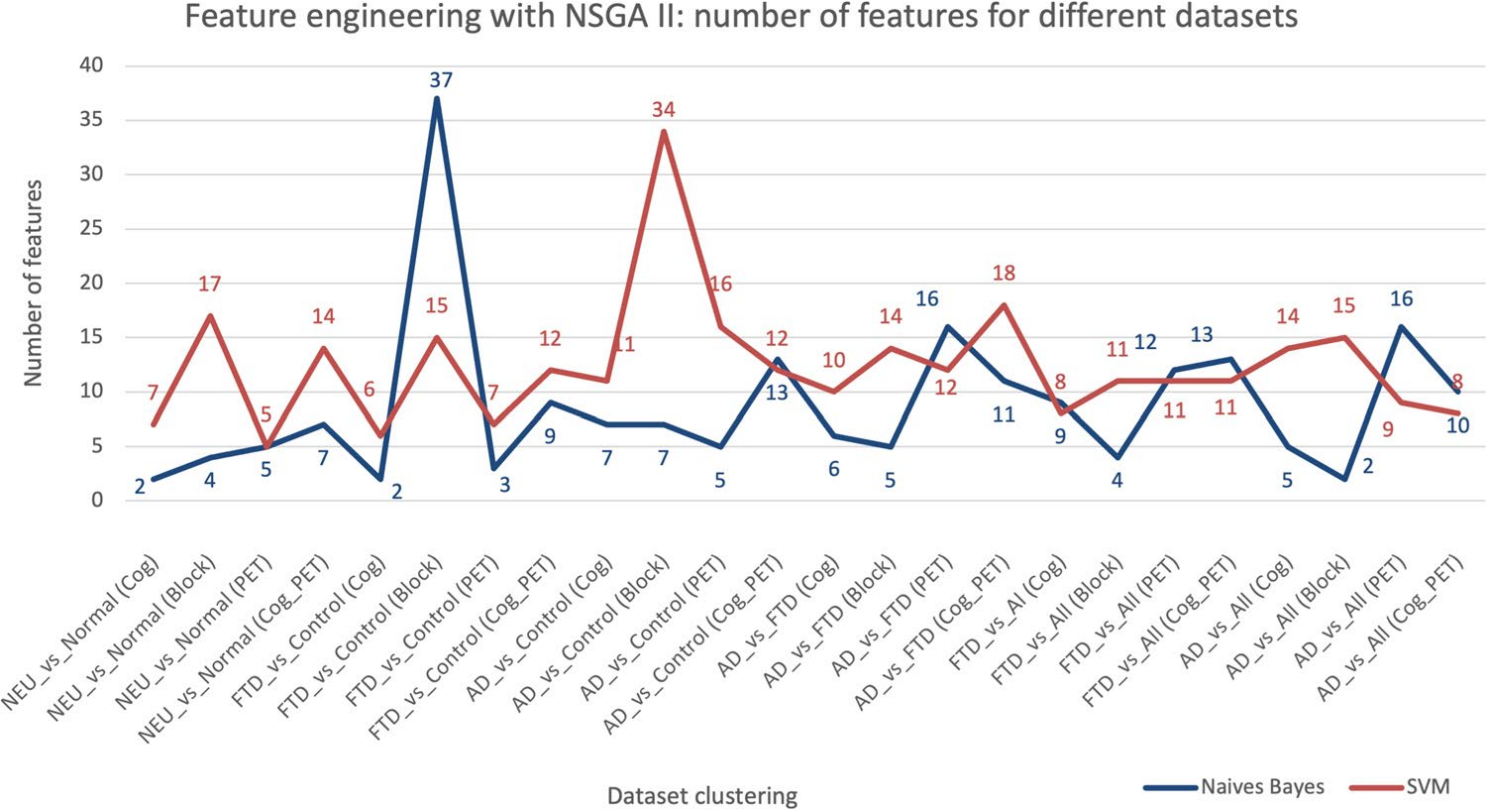
Comparative results of the Feature engineering using NSGAII, Naive Bayes and SVM algorithms as the fitness function. X axis represents the experiments addressed, and Y axis is the number of features obtained. Block means Demographic data + Cognitive test data without separating the scores associated with the same test

The NSGAII MOEAs obtain several solutions as part of the Pareto Front. The set of features selected for each potential solution can be visualized by the physicians to validate the clinical impact. Figure 8 shows results for several datasets: Demographic, Cognitive Test, and Brain Metabolism Data. Table 3 shows the features selected with SVM as fitness function for AD and FTD vs HC. In this example, Bayesian classifiers obtain the best results, with an average reduction of features of 91.52*%* compared to 87.97*%* for SVMs classifiers. Considering that the reduction percentage reached for both classifiers is really high, it is necessary to evaluate the performance each individual with Bayesian and SVMs classifiers as fitness function.

**Table 3:** *AD* − *FTD* vs HC: Features selected using NSGA II with SVM as fitness function for Cognitive, Block, PET and *PET* +Cognitive

Cognitive	Block	PET	*PET* +Cognitive
ace3_fluidity	education_years	o1_l	f1m_l
rocf_type_3min_4	cbtt_direct	brodmann_47	brodmann_35
mst_direct	cbtt_indirect	f1m_l	pcl_r
tmt_a	tmt_a	brodmann_35	sma_l
fcsrt_lt	tmt_b	brodmann_37	put_r
rocf_30min	fcsrt_l1		cau_l
education_years	fcsrt_lt		brodmann_19
	fcsrt_total		fcsrt_dif_free
	fcsrt_dif_free		ace3_total
	fcsrt_dif_total		fcsrt_dif_total
	addensbrook		sdmt
	ace3_total		rocf_type_copy_4
	ace3_attention		rocf_type_3min_7
	ace3_memory		rocf_type_copy_3
	ace3_fluidity		
	ace3_language		
	ace3_visospatial		

The solutions provided by the feature engineering approach are fed to the ML-based phase: classifiers and meta-model using EG as in Fig. 1, described in Section 3. For each problem, only one of the solutions provided in the feature selection phase has been selected for testing. Accuracy and F1-score as more qualified metrics have been selected for the analysis. We remind the reader that this work does not focus on the clinical analysis but on the possibilities opened by the developed tool. Hence, selected problems from the case of study will be presented in order to evaluate such capabilities of the computational tool.

**Fig. 9:**
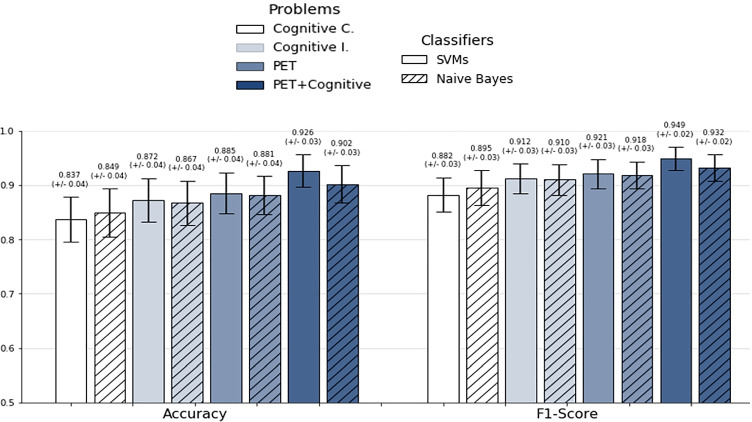
Classification performance achieved by one of the best feature subsets given by the NSGAII for each of the algorithms used to evaluate the fitness, when individuals with AD, FTD and healthy controls were evaluated. *Cognitive C.* denotes groupings of scores from the same cognitive test; *Cognitive. I.* considers each of the scores independently

As a result of the evaluation tests, the SVMs classifiers performed slightly better than the Bayesian classifiers as shown in Fig. 9. Table 4 presents the average values of the metrics used to assess the solutions from the Pareto front obtained with NSGA II, and applied in this case of study. High values of sensitivity and specificity indicate the reliability in predicting positive and negative cases, respectively. The significance of these results is evaluated using the *p* − *value* in Table 5. Very small *p* − *values* confirm the reliability of the study. According to the results, our ML-based tool is able to clearly differentiate between individuals with AD, FTD and healthy controls, especially when PET data are provided. A slightly lower performance is obtained working with cognitive dataset. Although cognitive test performance is closely associated with the brain metabolism of some regions, not all brain regions are covered during the neuropsychological examinations [16, 31, 37]. In addition, other factors such as cognitive reserve may limit the diagnostic capacity of neuropsychological examination in some cases [51].

**Table 4:** Pareto front assessment resulting from NSGA II

Dataset	Accuracy	Precision	Sensitivity	Specificity	F1−score
Naive Bayes					
Cognitive C.	0.849	0.914	0.879	0.766	0.895
Cognitive I.	0.867	0.908	0.913	0.741	0.910
PET	0.881	0.919	0.918	0.782	0.918
* PET* + *Cognitive*	0.902	0.954	0.912	0.875	0.932
Support Vector Machine					
Cognitive C.	0.837	0.941	0.832	0.852	0.882
Cognitive I.	0.872	0.927	0.898	0.800	0.912
PET	0.885	0.919	0.924	0.782	0.921
* PET* + *Cognitive*	0.926	0.966	0.933	0.906	0.949

**Table 5:** *P*−value for metrics applied to assess results from NSGA II

	*P*−value				
Dataset	Accuracy	Precision	Sensitivity	Specificity	F1−score
Naive Bayes					
Cognitive Ċ	3.02E-25	1.83E-16	2.76E-21	1.50E-15	9.14E-25
Cognitive I.	1.14E-27	2.27E-24	2.81E-22	3.82E-23	9.54E-28
PET	2.19E-28	1.25E-27	1.72E-19	1.91E-26	9.72E-28
* PET* + *Cognitive*	7.10E-29	3.72E-22	3.52E-23	8.59E-21	3.88E-28
Support Vector Machine					
Cognitive C.	3.19E-28	1.37E-13	5.43E-27	8.74E-13	3.84E-27
Cognitive I.	3.41E-27	1.42E-18	2.71E-25	3.54E-17	1.65E-27
PET	2.17E-26	3.80E-27	6.16E-19	1.91E-26	7.95E-26
* PET* + *Cognitive*	1.67E-22	6.19E-14	4.91E-19	1.12E-13	2.63E-22

**Fig. 10:**
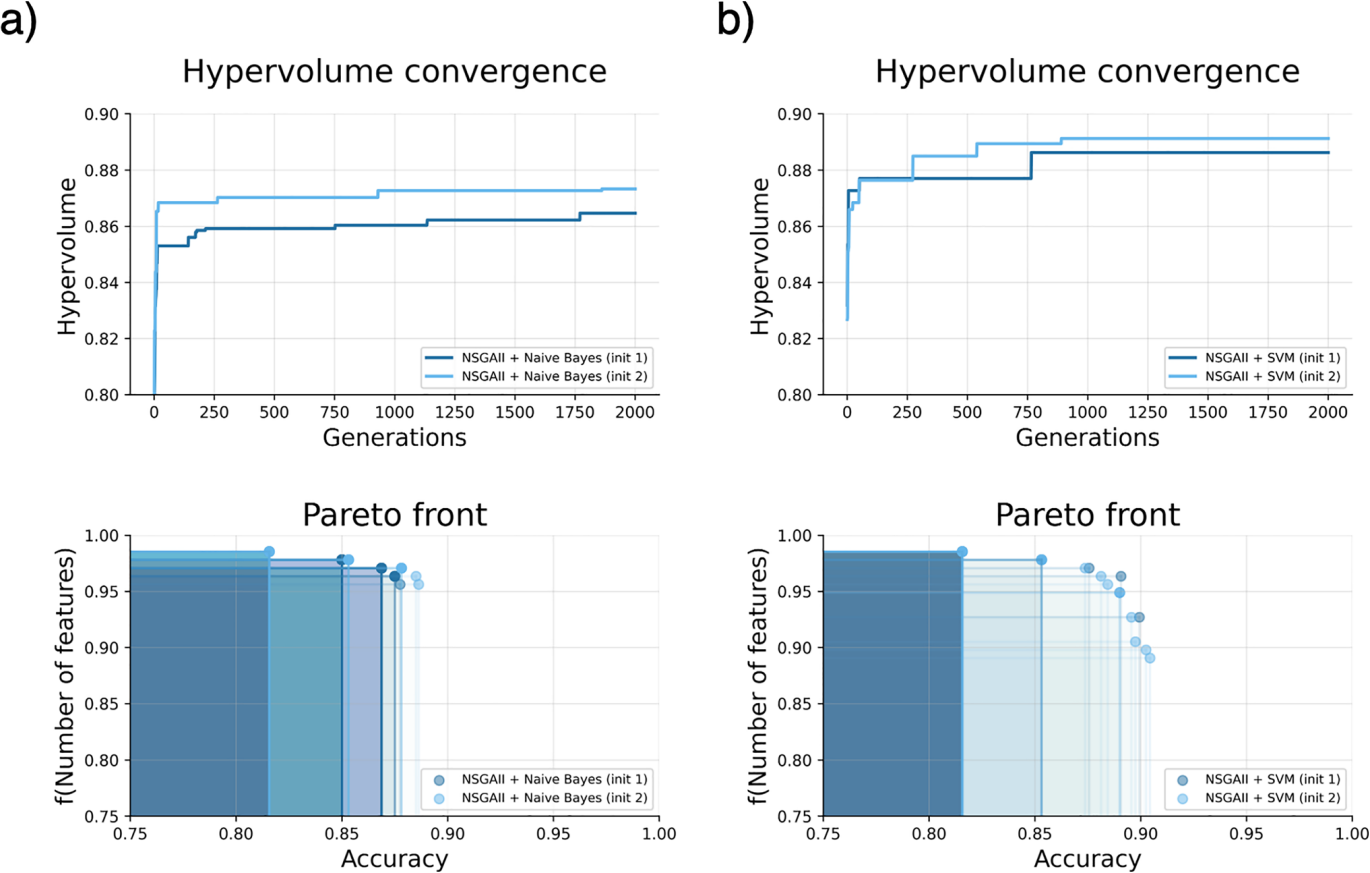
Convergence of the NSGAII for the Neurodegenerative Disorders (NEU) vs Healthy Controls (HC) diagnosis including PET data using (**a**) Naive Bayes classifier or (**b**) SVMs. NEU represents AD or bvFTD disorders. The pareto front subfigure is defined by equation: $1-\frac{\mathrm{Length}\;(\mathrm{Individual})}{Num.\;Features}$. The results of two different initialisations are shown

Moreover, this tool provides information about the evolution of the feature engineering process by means of a graphical representation of the evolution of the convergence of the MOEAs. For instance and related to the case of use, Fig. 10 represents the MOEA convergence for the PET datatests addressed using Naive Bayes and SVMs.

**Fig. 11:**
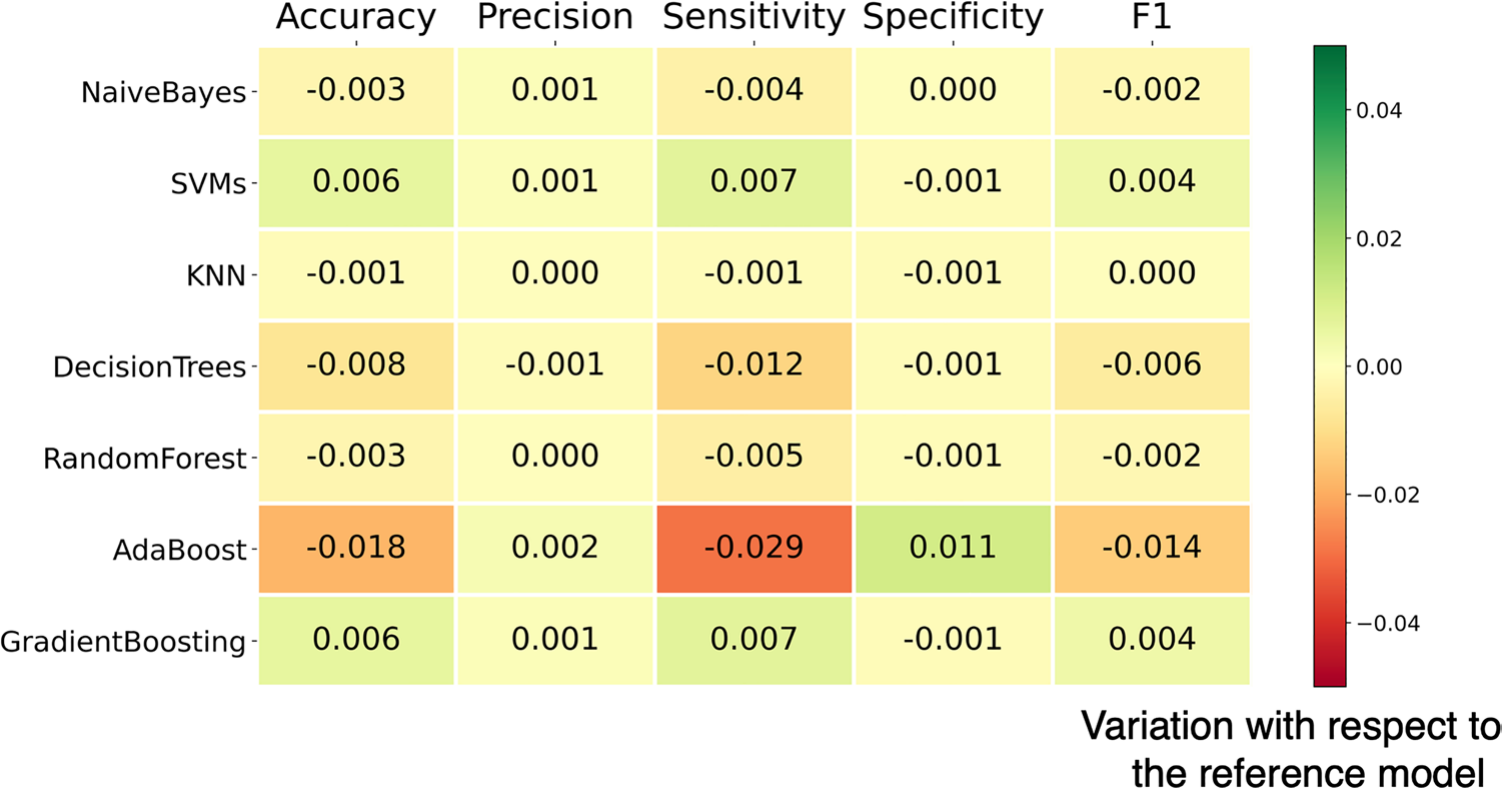
Variation in classification performance for the NEU (AD or bvFTD) vs HC diagnosis and PET data. The reference values *Acc* = 0.885, *Pre* = 0.919, *Rec* = 0.924, *F*1 = 0.921 correspond to the highest scores in Fig. 10

In addition, this tool also supplies different visual support representations to evaluate the performance of the classifiers. It implements the receiver operating characteristic (ROC), the confusion matrix, and a comparative graphical representation of the variation in classification performance among the different classifiers with respect to the best result obtained and with the best feature subset during the featured engineering phase (Fig. 9). Fig. 11 shows the variation performance for the case of use, where the hyperparameters were adjusted using a grid search strategy. Particularly, regularization parameters *λ* and *C* for SVM, the loss function (binomial deviance or exponential), the percentage of examples used to train the base models of the ensemble 3 and the number of characteristics 4. SVM and Gradient Boosting obtained the best performance with *F*1 − *score* = 0.925, although the rest of algorithms also reached high values. Table 6 shows the p-values computed from the performance metrics along all iterations. It can be seen that the obtained values are much smaller than 0.05 and we can state that the results are significant.

**Table 6:** *P*-value for metrics applied in the case study

P-value					
Classifier	Accuracy	Precission	Sensitivity	Specificity	F1-Score
Naive Bayes	1.95E-25	1.08E-24	4.05E-17	8.25E-23	6.42E-25
SVM	3.20E-25	2.35E-24	1.96E-17	1.30E-22	3.47E-25
KNN	2.16E-22	1.32E-23	5.38E-16	1.30E-22	4.04E-22
Decision Trees	4.59E-25	3.54E-24	7.41E-19	1.30E-22	1.02E-24
Random Forest	9.36E-25	1.37E-24	2.09E-15	1.30E-22	4.11E-24
Gradient Boosting	3.20E-25	2.35E-24	1.96E-17	1.30E-22	3.47E-25

**Fig. 12:**
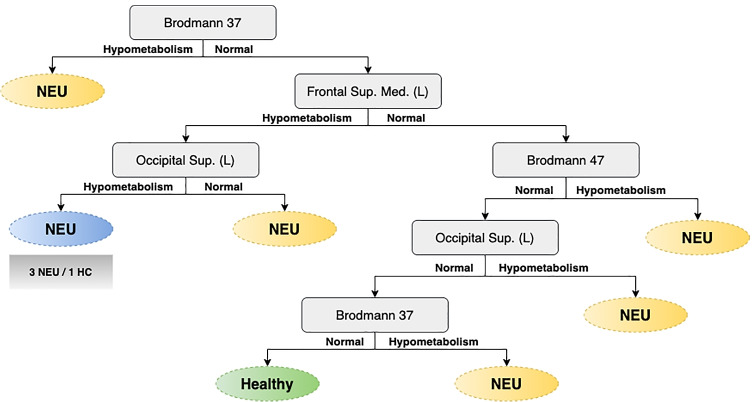
Decision trees corresponding to the classification problem NEU (AD or bvFTD) vs HC is presented as an example of a more interpretable graph. (Performance: Acc = 0.885 ± 0.04;Pre = 0.919 ± 0.03;Rec = 0.924 ± 0.04;F1 = 0.921 ± 0.03). Squares and ellipses represent nodes and leaves, respectively. The color blue denotes that most instances belong to the class indicated on the leaf but at least 1/4 correspond to the opposite class

One of the most important challenges for the clinical experts is the interpretability of the models. In this light, decision tree models have been developed to provide this capability to clinicians. This kind of algorithms provide a clear and simple set of rules that allow to distinguish between different clinical conditions. Figure 12 represents the decision tree for the case of use that we are presenting to give insights about the functionalities of this framework. This result was validated by expert neurologists who agreed on the clinical significance. According to the expert neurologists, in the decision tree, several key areas in the pathophysiology of AD and/or FTD are included. Specifically, regions in the frontal lobe (frontal superior medial gyrus and inferior frontal gyrus/Brodmann area 47), the temporal cortex (Brodmann area 37) and occipital lobe. According to the tree, the hypometabolism of any of these areas suggests the presence of a neurodegenerative disorder, while a normal metabolism in all areas is required to be classified as control.

### Meta-models

**Fig. 13:**
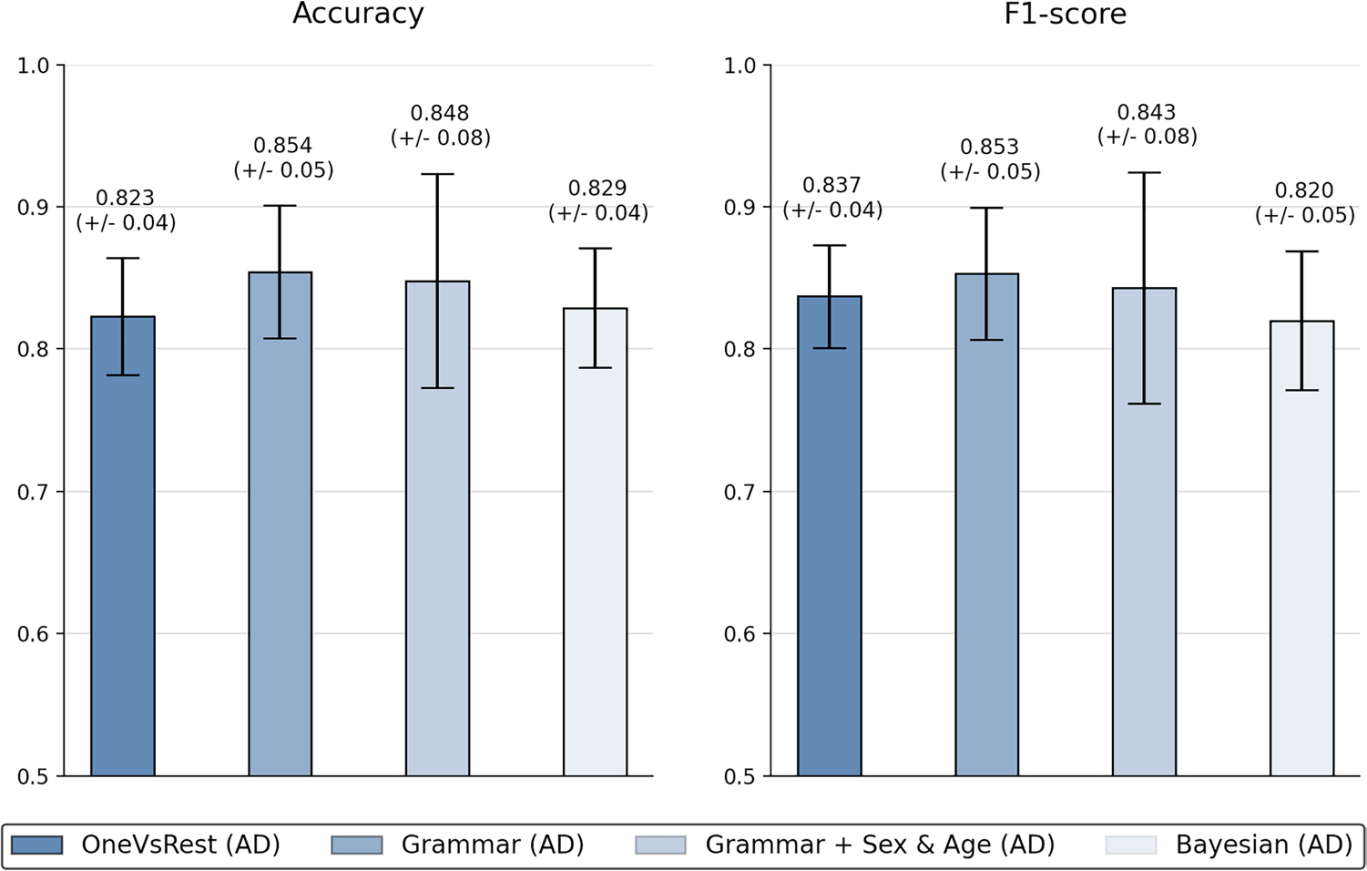
Performance obtained in multi-class classification integrating the output binary classifiers into a multiclass output. Ten repetitions of 5 CV were applied for the validation process of the reference model and the modeling strategy with Bayesian networks (described in Section 3.3.2)

This meta-model was designed to work with independent cognitive tests scores. The result is a multiclass classification model, which integrates the output of the binary classifiers using EG or Bayesian classifiers. In order to validate this module, we show the results obtained using the one vs rest classification models with the AD condition. Figure 13 shows the results obtained using accuracy and F1-score as metrics to evaluate the performance. It is clearly observed that the strategy with EG improved the classification results compared to the best results obtained using the binary classifiers independently and Bayesian networks. Even after including gender and age variables, which produce a loss of performance, EG overcomes the performance of the previous ones.

Results from this model with EG have demonstrated a great potential to improve the classification accuracy with limited datasets, as cognitive assessments.

## Conclusions

This paper has presented the design and implementation of a machine learning–based framework for the automatic diagnosis, especially, of neurodegenerative diseases. Neuropsychological and neuroimaging assessments provide large, heterogeneous datasets, with high possibilities for knowledge mining and the development of diagnostic tools. Our tool is proposed under the XAI perspective to support the clinicians in the diagnosis, as it provides all the steps required to analyse these datasets, from the data preprocessing, feature selection through an evolutionary approach, and modeling of the mentioned diseases.

As a case of study, we have evaluated the performance of our approach in the diagnosis of two widespread neurodegenerative diseases, AD and FTD. It was clearly observed how the proposed framework allows a smooth processing of the cognitive and image assessments, with a high reduction in the number of features needed for the diagnosis, and a high accuracy in the classification. A strong effort has been put on the interpretability of the results, showing how a data-centric point of view helps to understand AD and FTD disorders.

## Supplementary Information

Below is the link to the electronic supplementary material.

Supplementary file1 (PDF 373 KB)

## Data Availability

Al data are available in a systematic database created by the Department of Neurology of the San Carlos Hospital, in Madrid, and accessible to clinicians and researchers participating in the project. These data are not publicly available due to data privacy laws.

## Footnotes

1 Data that support this study are available on request from researchers

2 Supplementary Material is provided for this multiclass meta-model

3 This parameter generates a behavior analogous to bagging helping to reduce the variance of the model

4 This refers to the attributes used to train each of the base classifiers. This parameter generates a behavior similar to randomization. The parameters taken were $\sqrt {num\_features}$ or $\log _{2}(num\_features)$

## Code availability

Code is available in PyPi and GitHub
