# Supplementary material for: GA-MADRID: design and validation of a machine learning tool for the diagnosis of Alzheimer’s disease and frontotemporal dementia using genetic algorithms
Source: Med Biol Eng Comput. 2022 Jul 19;60(9):2737–56. doi: 10.1007/s11517-022-02630-z (PMC9365756; doi:10.1007/s11517-022-02630-z)
Supplement: Supplementary file 1 — Supplementary file1 (PDF 373 KB) [file 11517_2022_2630_MOESM1_ESM.pdf]

Supplemental Materials: GA-MADRID: Design and validation of a machine learning tool for the diagnosis of neugodegenerative disorders using genetic algorithms

1 Database structuring

This supplementary material contains information about the structure of the database designed for the proposed framework. The MySQL database schema is shown in the main document, and Figure 1 details the number of features in each table, the type of data and its description. Tables brodmann_qualitative / quantitative and aal_qualitative / quantitative, corresponding to the brain metabolism. References Bitam and Mellouk (2006); Tzourio-Mazoyer et al. (2002) presented the two different atlases considered in this framework, the Brodmann's atlas (47 regions) and the Automated Anatomical Labelling (AAL) atlas (90 regions), respectively.

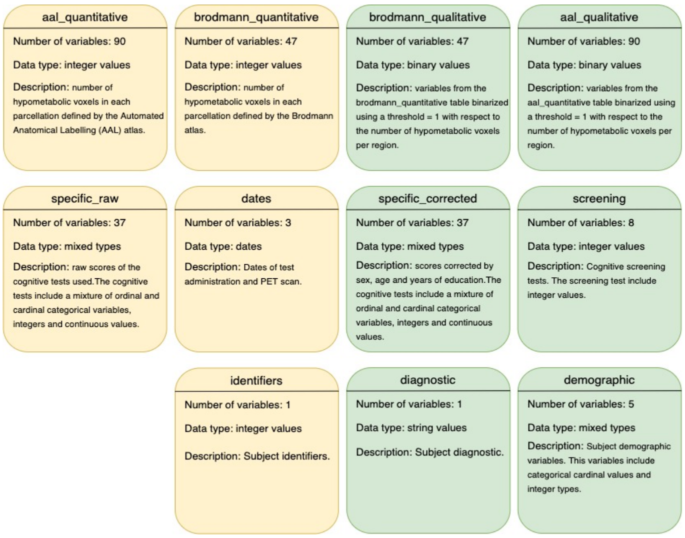

Fig. 1: Structure of the database used in the framework. Tables used in the modeling tasks are highlighted in green. The schema defines the number of variables in each table, the type of data it contains and the description of the data. The brain atlases used to define the brain regions of the tables aal_quantitative, aal_qualitative; and brodmann_quantitative and brodmann_qualitative; correspond to Bitam and Mellouk (2006) and Tzourio-Mazoyer et al. (2002) respectively.

2 Metamodel with Bayesian network

This part of the supplementary material explains in more detail our multiclass metamodel proposal using Bayesian Networks. With a model based on two layers,

the first one consists on the result of applying binary classifications to different problems. A problem is defined according to the dataset used and, each dataset contains a different set of features, which are selected after the feature engineering process implemented in this computational tool. This first version defines six different binary classifiers to predict AD, FTD or HC, which results are the positive class (1) or negative class (0). These binary classifiers are listed below:

1. **FTDvsHC**. Positive class: FTD; Negative class: HC.
2. **ADvsHC**. Positive class: AD; Negative class: HC.
3. **NEUvsHC**. Positive class: FTD, AD; Negative class: HC.
4. **ADvsFTD**. Positive class: AD; Negative class: FTD.
5. **FTDvsADandHC**. Positive class: FTD; Negative class: AD, HC.
6. **ADvsFTDandHC**. Positive class: AD; Negative class: FTD, HC.

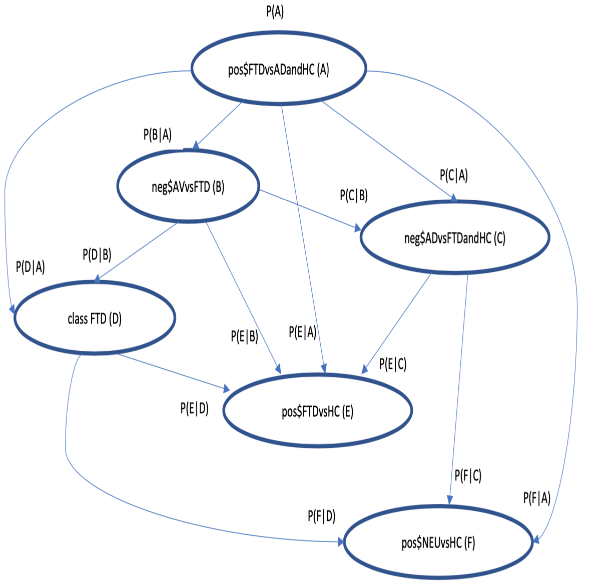

Fig. 2: DAG as an example result of the two layers meta-model proposed, where nodes are the binary classifiers for each problem and arcs depends on the distribution probabilities. P(X—Y) represents the distribution probability of reaching the node via that path.) Bitam and Mellouk (2006) and Tzourio-Mazoyer et al. (2002) respectively.

The second layer consists on the Bayesian Network that receives the binary classifiers results. Bayesian networks represent the joint probability distribution of the variables defined, and do Bayesian inference with these variables. According to the graph theory, a directed graph is a pair (V,E), where V is a finite, nonempty set of elements called nodes or vertices, and E is an ordered set of elements of V, called edges or arcs. A directed acyclic graph (DAG) is a directed graph, which does not contain directed cycles. Bayesian Networks represent the joint probability

by DAGs and, in a very simplified way, it illustrates if the Markov condition is satisfied or not. Figure 2 shows one of the DAG resulting of the multiclass meta-model. As a result of the Bayesian Network, the result is the class with the highest probability.

References

Bitam, S., Mellouk, A., 2006. Brodmann's Localisation in the Cerebral Cortex. Springer. chapter Description of individual brain maps. p. 298.

Tzourio-Mazoyer, N., Landeau, B., Papathanassiou, D., Crivello, F., Etard, O., Delcroix, N., Mazoyer, B., Joliot, M., 2002. Automated anatomical labeling of activations in spm using a macroscopic anatomical parcellation of the mni mri single-subject brain. NeuroImage 15, 273 – 289. doi:https://doi.org/10.1006/nimg.2001.0978.
